# No Interference of H9 Extract on Trastuzumab Pharmacokinetics in Their Combinations

**DOI:** 10.3390/ijms242316677

**Published:** 2023-11-23

**Authors:** Seung Yon Han, Jeong-Eun Yu, Byoung Hoon You, Seo-Yeon Kim, Mingoo Bae, Hee-Sung Chae, Young-Won Chin, Soo-Hwa Hong, Ju-Hee Lee, Seung Hyun Jung, Young Hee Choi

**Affiliations:** 1 College of Pharmacy and Integrated Research Institute for Drug Development, Dongguk University_Seoul, 32 Dongguk-ro, Ilsandong-gu, Goyang-si 10326, Gyeonggi-do, Republic of Korea; hsyglory@gmail.com (S.Y.H.); yyy0982@naver.com (J.-E.Y.); hoon4131@nate.com (B.H.Y.); kimsy7856@naver.com (S.-Y.K.); nophra88@naver.com (M.B.); hchae@olemiss.edu (H.-S.C.); 2 National Center for Natural Products Research, School of Pharmacy, The University of Mississippi, Oxford, MS 38677, USA; 3 College of Pharmacy and Research Institute of Pharmaceutical Sciences, Seoul National University, 1, Gwanak-ro, Gwanak-gu, Seoul 08826, Republic of Korea; ywchin@snu.ac.kr; 4 Department of Korean Internal Medicine, Dongguk University Bundang Korean Medicine Hospital, Seongnam-si 13601, Gyeonggi-do, Republic of Korea; hongsh9646@gmail.com; 5 College of Korean Medicine, Dongguk University, Gyeongju-si 38066, Gyeongsangbuk-do, Republic of Korea; jh1548@dongguk.ac.kr (J.-H.L.); omdjeong@naver.com (S.H.J.)

**Keywords:** trastuzumab, H9 extract, pharmacokinetic interaction, combination, dosage regimen

## Abstract

Trastuzumab is used to treat breast cancer patients overexpressing human epidermal growth factor receptor 2, but resistance and toxicity limit its uses, leading to attention to trastuzumab combinations. Recently, the synergistic effect of trastuzumab and H9 extract (H9) combination against breast cancer has been reported. Because drug exposure determines its efficacy and toxicity, the question of whether H9 changes trastuzumab exposure in the body has been raised. Therefore, this study aimed to characterize trastuzumab pharmacokinetics and elucidate the effect of H9 on trastuzumab pharmacokinetics at a combination dose that shows synergism in mice. As a result, trastuzumab showed linear pharmacokinetics after its intravenous administration from 1 to 10 mg/kg. In the combination of trastuzumab and H9, single and 2-week treatments of oral H9 (500 mg/kg) did not influence trastuzumab pharmacokinetics. In the multiple-combination treatments of trastuzumab and H9 showing their synergistic effect (3 weeks of trastuzumab with 2 weeks of H9), the pharmacokinetic profile of trastuzumab was comparable to that of 3 weeks of trastuzumab alone. In tissue distribution, the tissue to plasma ratios of trastuzumab below 1.0 indicated its limited distributions within the tissues, and these patterns were unaffected by H9. These results suggest that the systemic and local exposures of trastuzumab are unchanged by single and multiple-combination treatments of H9.

## 1. Introduction

The human epidermal growth factor receptor 2 (HER2 or HER-2/neu) is overexpressed in 14–20% of breast cancer patients. The intratumor heterogeneity of HER2 is associated with aggressive tumor growth, high relapse rates and poor survival [1,2,3,4]. Trastuzumab, a 150 kDa monoclonal antibody targeting HER2, is popularly used for the treatment of HER2-positive breast cancers [5,6,7,8]. Trastuzumab resistance and trastuzumab-induced cardiotoxicity (TIC) sometimes limit the clinical uses of trastuzumab and can cause cancer recurrence [9,10,11].

Two antigen-specific sites of trastuzumab bind to the HER2 extracellular domain, and block the cleavage of the extracellular domain of HER2 and the formation of HER2 dimerization. This mechanism suppresses the proliferation of HER2-overexpressed tumor cells by blocking downstream signaling pathways including MAPK/PI3K/AKT [9,12,13,14]. The PI3K/AKT/mTOR pathway, as a downstream signaling pathway of HER2–trastuzumab interaction, is also closely associated with trastuzumab resistance [15,16]. Continual trastuzumab treatment inhibits the cleavage of the extracellular domain of HER2 and the formation of HER2 dimerization. Consequently, this weakens the HER2 signaling pathway that inhibits PI3K/AKT/mTOR, thereby leading to trastuzumab resistance [1,2,3,4,15,16]. A truncated mutant HER2 (i.e., a variant of the common structure of HER2) leads to the overexpression of HER3 receptors as an alternative mechanism to compensate for HER2-mediated signaling pathways. The overexpressed HER3 receptors activate the PI3K/AKT pathway and cause trastuzumab resistance in breast cancers [17]. The overexpression of HER3 receptors by trastuzumab even happens in the absence of influencing HER2 [17,18]. In other words, the sufficient blocking of the abnormally activated or maintained PI3K/AKT/mTOR pathway can reverse drug resistance against tumors for successful trastuzumab therapy [15,16,19,20]. In addition, TIC, as the major adverse effect of trastuzumab, has been noted in trastuzumab monotherapy because it occurs through the same pathway, promoting cytotoxicity and apoptosis in cancer cells. The blocking of HER2-mediated PI3K/AKT pathways by trastuzumab causes oxidative stress and apoptosis in cardiomyocytes [9,10,11,14,15,16]. Thus, the regulation of different targets to prevent and/or attenuate TIC is required [21]. For example, AMPK activation has emerged as a potential solution to regulate TIC in accordance with the optimization of trastuzumab-dosage regimens [9,14].

The combination of chemotherapy with Oriental medicines has become attractive, as multiple components of Oriental medicines possess various pharmacological activities. Studies on combinations of trastuzumab with Oriental medicines have reported synergistic efficacy as well as reduced cancer recurrence, drug resistance and side effects [9,15,22,23,24,25]. H9 extract (H9) is a formula of nine medicinal herbs derived from a traditional Korean prescription of Osuyubujaijung-tang, which possesses antioxidant and cytoprotective effects and ameliorates the symptoms of peripheral T-cell lymphoma [20,26,27]. The nine Oriental medicinal herbs exhibiting anti-cancer activities compose of H9 as follows: *Panax ginseng* C. A. Mey. (20%); *Cinnamomum cassia* Blume (12%); *Evodia rutaecarpa* (Juss.) Benth. (8%); *Foeniculum vulgare* Gaertner (12%); *Psoralea corylifolia* Linne (12%); *Myristica fragrans* Houtt. (12%); *Alpinia officinarum* Hance (8%); *Sparganium stoloniferum* Buchanan- Hamilton (4%); and *Curcuma longa* Linne (12%) [20]. Several volatile compounds and their relative percentages in H9, such as coumarin (20.27%), isoeugenol (13.16%), methylisoeugenol (5.0%), methoxy isoeugenol (23.13%), isoelemicin (3.126%), and angelicin or psolarene (12.35% and 14.15%), were also identified [28]. Among them, anti-cancer activities of coumarin and angelicin were considered to potentially contribute to the anti-cancer effect of H9 [29,30]. Even though the active constituents in H9 and their pharmacological activities have not been definitely identified with some of them remaining unknown, the anticancer activities of H9 against a lung cancer model and breast cancer models have been observed [28,31,32]. H9 inhibits the proliferation of breast cancer cells by inhibiting HER2-PI3K/AKT and activating AMPK, resulting in G0/G1 arrest and subsequent apoptosis [20,33]. The AMPK activation regulates HER2 and the epidermal growth factor receptor (EGFR) in HER2-amplified breast cancer cells [34], leads to myocardial protection and attenuates TIC [35,36]. From this perspective, H9 is expected to accelerate HER2-mediated PI3K/AKT inhibition and ameliorate TIC through AMPK activation. Already, these dual-targeting mechanisms of H9 combined with trastuzumab exerted stronger anti-cancer activity by inhibiting PI3K/AKT and activating AMPK than did either drug individually, producing a synergistic effect by inducing apoptosis in mice bearing a breast tumor with combination index (CI) values less than 1 (CI = 0.647) [20,32,37,38].

**Figure 1:**
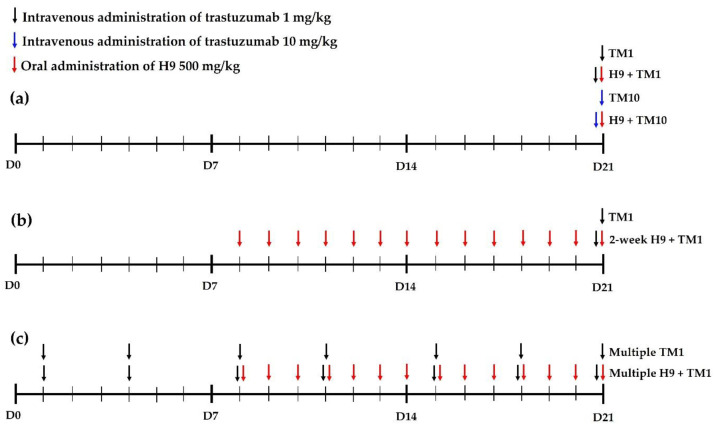
Dosage design scheme of trastuzumab and H9 according to the experimental groups of this study: (**a**) 1 and 10 mg/kg with single treatment of trastuzumab with or without H9, (**b**) 1 mg/kg with single treatment of trastuzumab with or without multiple treatment of H9, (**c**) 1 mg/kg with multiple treatment of trastuzumab with or without multiple treatment of H9.

According to dosage regimens (i.e., dose, dosing interval and dosing period) in combination therapy, a perpetrator drug (e.g., H9) can influence the pharmacokinetic profile of a victim drug (e.g., trastuzumab), which determines efficacy and/or toxicity of a victim drug [39,40]. Despite the synergistic potency of the trastuzumab and H9 combination, there is currently no information available on the pharmacokinetics of trastuzumab with H9 [20]. Therefore, we characterized trastuzumab pharmacokinetics after its intravenous administration at 1 and 10 mg/kg with single and multiple treatment and elucidated the effect of H9 on the pharmacokinetics of trastuzumab (Figure 1).

## 2. Results

### 2.1. Dilution Linearity for Trastuzumab

The dilution effects for trastuzumab levels in plasma and various tissues are listed in Table 1. Because the range of calibration standards provided with the enzyme-linked immunosorbent assay (ELISA) kit was 0.1–3 µg/mL, dilution was required for trastuzumab concentrations greater than 3 µg/mL in plasma or various tissue samples. The dilution factors were determined using five blank mouse-plasma samples spiked with trastuzumab. Plasma samples were diluted at 1:40, 1:20, 1:10, and 1:2 in assay buffer to a final concentration for 0.1 and 1 µg/mL (n = 5 for each dilution). For each dilution, the accuracy ranged from −0.81% to 3.96% for 0.1 μg/mL and from −2.99% to 4.87% for 1 µg/mL (Table 1). This result suggested that there was no interference when the plasma samples were diluted up to 40-fold.

The dilution factors of mouse tissue homogenates were also determined at 1:2 (for gastrointestinal tract (GI)), and 1:10, 1:20 and 1:40 dilution (for all tissues) in assay buffer. Each tissue sample was diluted to a final concentration of 0.1 and 1 µg/mL (n = 5 for each dilution). For each dilution, the accuracy ranges at 0.1, and 1 µg/mL in each tissue were as follows (Table 1): −3.79% to 3.51%, and 1.80% to 5.09% in the GI; −4.78% to 3.65%, and −3.19% to 2.31% in the lung; −3.04% to 0.924%, and 0.801% to 4.10% in the liver; 2.85% to 9.23%, and −2.79% to 3.58% in the spleen; −1.28% to 5.69%, and −0.0202% to 5.31% in the muscle; 0.167% to 6.88%, and 0.845% to 3.71% in the heart; and −0.230% to 2.98%, and 2.78% to 3.63% in the kidney, respectively. These results suggested that there were no interferences when tissue samples were diluted up to 40-fold. 

### 2.2. ELISA Validation

The precision and accuracy of inter- and intra-day measurements of trastuzumab levels in plasma and various tissues are listed in Table 2. The inter- and intra-day precision and accuracy were used to evaluate the efficiency of the sandwich ELISA method by measuring different concentrations of quality control (QC) samples of trastuzumab (0.1, 0.3, 1 and 3 µg/mL). This ELISA method was performed according to the US FDA bioanalytical method validation guidance for industry [41]. The inter-day precision and accuracy assessments were based on the measurement of trastuzumab levels for five independent batches. The range of the calibration curve was 0.1–3 µg/mL, and all samples had a correlation coefficient (R2) greater than 0.989. The lower limit of quantification (LLOQ) was 0.1 µg/mL. The accuracy of QC samples for plasma was within the range of −2.30% to 3.44%, and 0.339% to 2.50% for intra-day and inter-day measurements, respectively. The precision of QC samples for plasma was within the range of 5.15% to 7.23%, and 4.68% to 6.32% for intra-day and inter-day measurements, respectively. In tissues, the accuracy of QC samples for the GI, lung, liver, spleen, muscle, heart, and kidney was within the range of −2.20% to 6.31% (−2.18% to 4.16%) for intra-day (inter-day) validation, respectively. The precision of QC samples for the GI, lung, liver, spleen, muscle, heart, and kidney was within the range of 2.01% to 8.80% (3.63% to 8.02%) for intra-day (inter-day) validation, respectively. 

### 2.3. Stability Test

No significant degradation of trastuzumab standard samples occurred during short-term storage for 1 day, mid-term storage for 7 days, and long-term storage for 12 days at −80 °C, with ±15% deviation between the measured and nominal concentrations. This result indicated that trastuzumab was stable in mouse plasma for at least 12 days at −80 °C.

### 2.4. Trastuzumab Pharmacokinetics after Its Intravenous Administration (1 mg/kg) with and without Oral H9 (500 mg/kg) in Mice

**Figure 2:**
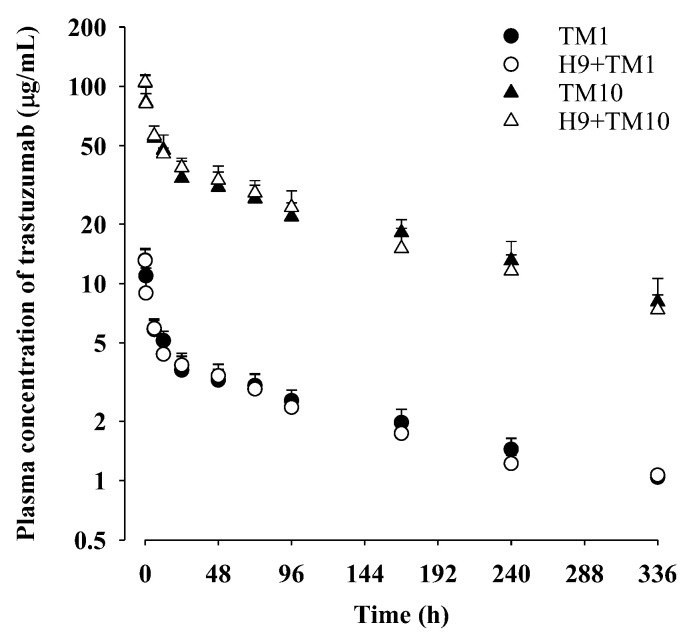
Mean (±SD) plasma concentrations of trastuzumab after intravenous administration of trastuzumab at a dose of 1 mg/kg with (○; n = 6) and without (●; n = 7) 500 mg/kg of oral H9 in mice. Corresponding values in trastuzumab at a dose of 10 mg/kg with (△; n = 6) and without (▲; n = 6) 500 mg/kg of oral H9 in mice. Error bars represent standard deviations.

The mean plasma concentration–time profiles of trastuzumab after its intravenous administration at a dose of 1 mg/kg with and without oral H9 at a dose of 500 mg/kg to mice are shown in Figure 2 and the relevant pharmacokinetic parameters are listed in Table 3. Also, the pharmacokinetic parameters calculated using a non-compartment model (Table S2) are listed in Table S3.

After intravenous administration of trastuzumab at a dose of 1 mg/kg, the plasma concentration of trastuzumab slowly decreased with a terminal half-life of 167 ± 37.0 h and a time-averaged total body clearance (CL) of 1.03 ± 0.116 mL/h/kg for the TM1 group. The apparent volume of distribution at a steady state (V_ss_) of trastuzumab was 233 ± 40.5 mL/kg for the TM1 group, which is slightly larger than the plasma volume of mice (50 mL/kg) [42,43]. Trastuzumab was detected in plasma for up to 336 h, and the contribution of the plasma concentration–time curve from the time of zero to the last blood sampling time (AUC_0–336 h_) was 72.8% of the plasma concentration–time curve from the time of zero to infinity (AUC_0–∞_) of trastuzumab in the TM1 group. After intravenous administration of trastuzumab with oral H9, all pharmacokinetic parameters of trastuzumab (e.g., AUC_0–336 h_, AUC_0–∞_, CL, terminal half-life, mean residence time (MRT) and V_ss_) in the H9+TM1 group were comparable to those in the TM1 group. These results indicated that oral H9 at a dose of 500 mg/kg might not have changed trastuzumab pharmacokinetics after its intravenous administration at 1 mg/kg in mice.

### 2.5. Trastuzumab Pharmacokinetics after Its Intravenous Administration (10 mg/kg) with and without Oral H9 (500 mg/kg) in Mice

The mean plasma concentration–time profiles of trastuzumab after its intravenous administration at a dose of 10 mg/kg with and without oral H9 at a dose of 500 mg/kg to mice are shown in Figure 2, and the relevant pharmacokinetic parameters are listed in Table 3. Also, the pharmacokinetic parameters calculated using the non-compartment model (Table S2) are listed in Table S3.

After intravenous administration of trastuzumab at a dose of 10 mg/kg, the plasma concentration of trastuzumab slowly decreased with a terminal half-life of 160 ± 30.0 h and CL of 1.20 ± 0.286 mL/h/kg in the TM10 group. The pharmacokinetic parameters such as terminal half-life, CL, V_ss_, and MRT were similar to those after intravenous administration of trastuzumab at 1 mg/kg. The AUC_0–336 h_ and AUC_0–∞_ of trastuzumab in the TM10 group, 6804 ± 1179 µg h/mL and 8737 ± 1952 µg h/mL, were approximately 10-fold higher than those after intravenous administration of trastuzumab at 1 mg/kg. These results indicated that trastuzumab pharmacokinetics after its intravenous administration in the range of 1 and to 10 mg/kg showed linear patterns in mice.

After intravenous administration of trastuzumab at a dose of 10 mg/kg with oral H9, all pharmacokinetic parameters of trastuzumab were comparable between the TM10 and H9+TM10 groups. This result indicated that oral H9 at 500 mg/kg might not have affected trastuzumab pharmacokinetics after its intravenous administration at 10 mg/kg. In addition, the AUC_0–336 h_ and AUC_0–∞_ values in the H9+TM10 group were approximately 10-fold higher than those in the H9+TM1 group, and other pharmacokinetic parameters (e.g., terminal half-life, CL and V_ss_) were comparable between the H9+TM10 and H9+TM1 groups.

### 2.6. Trastuzumab Pharmacokinetics after Its Intravenous Administration (1 mg/kg) with and without 2-Week Treatment of Oral H9 (500 mg/kg) in Mice

**Figure 3:**
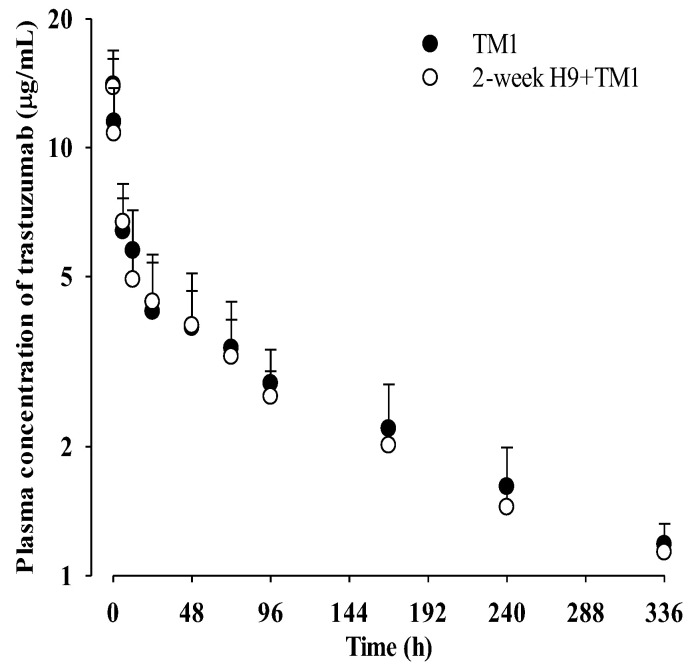
Mean (± SD) plasma concentrations of trastuzumab after its intravenous administration at a dose of 1 mg/kg with (○; n = 6) and without (●; n = 6) 2-week pretreatment of 500 mg/kg of oral H9 in mice. Error bars represent SD.

The mean plasma concentration–time profiles of trastuzumab after its intravenous administration at a dose of 1 mg/kg with and without 2-week treatment of oral H9 at a daily dose of 500 mg/kg in mice are shown in Figure 3, and the relevant pharmacokinetic parameters are listed in Table 4. Also, the pharmacokinetic parameters calculated using the non-compartment model (Table S2) are listed in Table S4. No significant difference in any of the pharmacokinetic parameters of trastuzumab were observed between the TM1 and 2-week H9+TM1 groups, indicating that 2-week treatment of oral H9 might not have changed trastuzumab pharmacokinetics in mice.

### 2.7. Trastuzumab Pharmacokinetics after Multiple Intravenous Administration of Trastuzumab (1 mg/kg) with and without Oral H9 (500 mg/kg) in Mice

**Figure 4:**
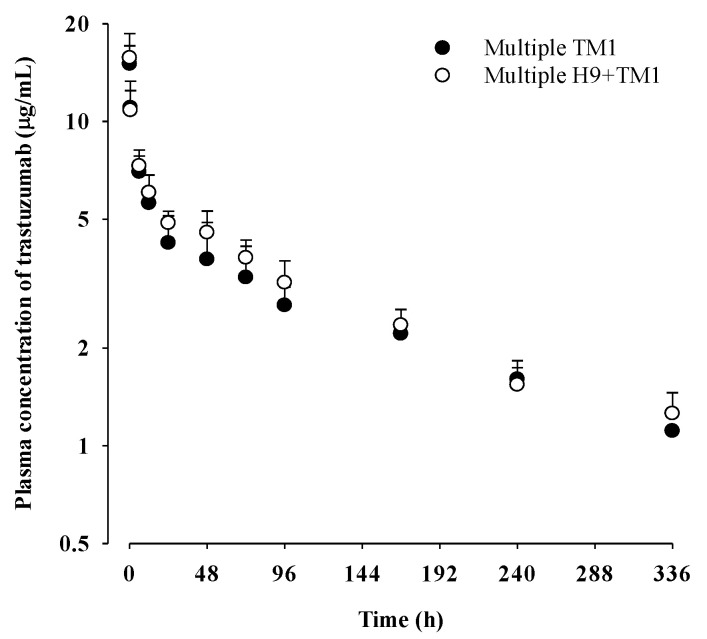
Mean (±SD) plasma concentrations of trastuzumab after its intravenous administration at a dose of 1 mg/kg with and without 500 mg/kg of oral H9 in multiple TM1 (○; n = 5) and multiple H9+TM1 mice (●; n = 6), respectively. Trastuzumab (1 mg/kg) was intravenously administered twice weekly for 3 weeks, and H9 (500 mg/kg) was orally administered once daily for 2 weeks in multiple H9+TM1 mice, whereas multiple TM1 mice were pretreated with the same dose and frequency of trastuzumab without 500 mg/kg of oral H9, respectively.

The mean plasma concentration–time profiles of trastuzumab after multiple intravenous administrations of trastuzumab at a dose of 1 mg/kg with and without oral H9 at a dose of 500 mg/kg to mice are shown in Figure 4, and the relevant pharmacokinetic parameters are listed in Table 5. Also, the pharmacokinetic parameters calculated using the non-compartment model (Table S2) are listed in Table S5. Compared to the pharmacokinetic parameters (e.g., AUC_0–336 h_, AUC_0–∞_, terminal half-life, CL and V_ss_) in the TM1 group, those in the multiple TM1 group were not significantly changed, suggesting that the twice-weekly intravenous administration of trastuzumab at a dose of 1 mg/kg for 3 weeks might not have accumulated in mice. In addition, the pharmacokinetic parameters of trastuzumab in the multiple H9+TM1 group were comparable to those in the multiple TM1 group, indicating that daily 500 mg/kg oral administration of H9 might not have changed trastuzumab pharmacokinetics in mice.

### 2.8. Tissue Distribution of Trastuzumab after Its Intravenous Administration (10 mg/kg) with and without Oral H9 (500 mg/kg) in Mice

**Figure 5:**
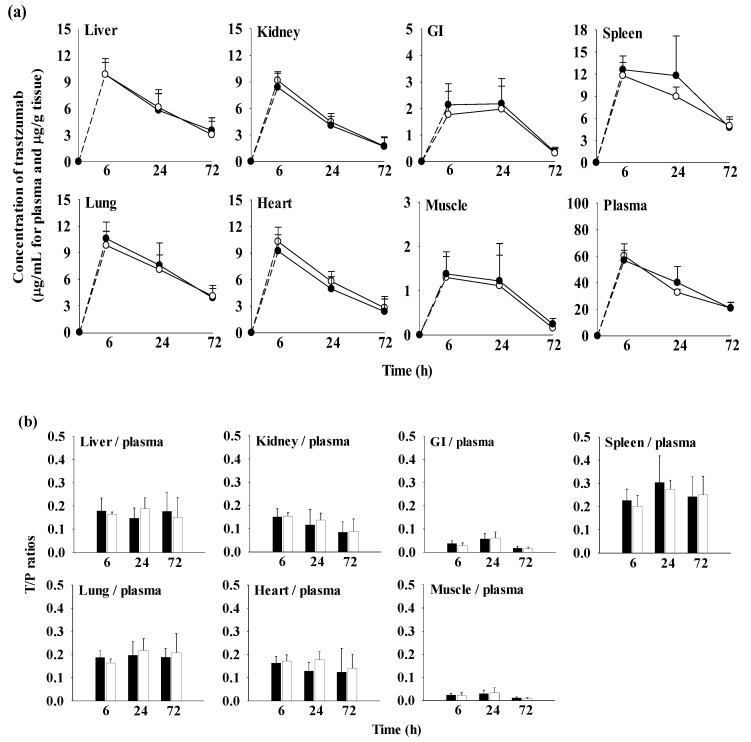
(**a**) Mean concentrations (μg/mL for plasma and μg/g tissue) and (**b**) T/P ratios of trastuzumab in plasma and tissues after intravenous administration of trastuzumab at a dose of 1 mg/kg with (○; n = 5) and without (●; n = 5) 500 mg/kg of oral H9 to mice. Error bars represent SD.

The quantitative distribution of trastuzumab in tissues such as the liver, heart, lung, spleen, kidney, GI, and muscle at 6 h (n = 5), 24 h (n = 5), and 72 h (n = 5) after intravenous administration of trastuzumab at a dose of 10 mg/kg with and without oral H9 at a dose of 500 mg/kg is shown in Table S1 and Figure 5. To further assess trastuzumab exposure in each tissue, the AUC_0–72 h_ values of trastuzumab were also calculated as shown in Table 6.

Although trastuzumab was detected in the liver, heart, lung, spleen, kidney, GI, and muscle up to 72 h, the concentration of trastuzumab in each tissue was significantly lower than that in the plasma. The tissue-to-plasma (T/P) ratios for all tissues in the TM10 and H9+TM10 groups were below 1.0, within the ranges of 0.0224 to 0.226 at 6 h, 0.0339 to 0.274 at 24 h and 0.00754 to 0.250 at 72 h, respectively. These results indicated that trastuzumab had a low affinity for tissues, which was supported by the considerably small V_ss_ values in the range of 230 to 254 mL/kg after the intravenous administration of trastuzumab in mice (Table 3). Among the tissues, the highest T/P ratio at each time was observed in the spleen (0.226 ± 0.0487 at 6 h, 0.304 ± 0.115 at 24 h, and 0.242 ± 0.0864 at 72 h in TM10, and 0.201 ± 0.0461 at 6 h, 0.274 ± 0.0399 at 24 h, and 0.250 ± 0.0807 at 72 h in H9+TM10).

Regarding the tissue exposure of trastuzumab, the AUC_0–72 h_ (µg h/g tissue) values of trastuzumab in all tissues were comparable between the TM10 and H9+TM10 groups.

## 3. Discussion

According to numerous reports, HER2 bound by trastuzumab blocks the PI3K/AKT pathway and inhibits the proliferation of cancer cells’ overexpressed HER2 receptors, and the combination of trastuzumab with H9 has the potential to synergize efficacy [20,32]. Co-treatment of trastuzumab with H9 delays tumor growth in tumor-bearing mice [32] and significantly inhibits the growth of BT-474 cells by inducing apoptosis. Considering that H9 inhibits HER2-PI3K/Akt pathways and activates AMPK, AMPK activation can provide a potential target in breast cancers [20,33]. AMPK activation can inhibit breast cancer cell’s growth and increase their sensitivity to chemotherapy and radiotherapy [44]. AMPK works in an opposite manner to the Akt pathway and negatively regulates the mTOR pathway, which has been correlated with tumor suppression. A more interesting finding by Gundewar et al. [35] is that AMPK activation by metformin provides significant myocardial protection through downstream signaling pathways involving eNOS and PGC-1. In line with this, the improvement of TIC using AMPK agonists (i.e., AICAR and metformin) has been proved in IPSC-CMS [36]. Thus, AMPK activation by H9 is expected to ameliorate TIC. It is of great clinical interest to investigate whether AMPK agonists can be used to combat trastuzumab resistance as well as TIC [9,20]. However, as far as we know, there is currently no information available on the pharmacokinetics of trastuzumab with H9 despite its synergistic potency [20]. Although the dosage regimen of the trastuzumab and H9 combination showed a synergistic effect which has already been investigated, how they move and interact with each other in the body remains unknown.

In the evaluation of drug–herb interactions, it is necessary to identify active constituent(s) in herbal products (similar to oriental medicines). A herbal product consists of numerous compounds possessing various pharmacological activities, and the interactions among constituents in the herbal product can happen. Nevertheless, numerous herbal products have been used on the condition that the active constituent(s) may not be definitely identified [45,46]. From this perspective, the FDA recognizes that there are technical challenges in determining the pharmacokinetics of a herbal product because a herbal product consists of more than one chemical constituent, and the active constituents may not be identified. It is acceptable to assess how a herbal product affects the pharmacokinetics of a combination drug, even if the reverse interaction, the effects of the herbal product, is not examined. In practice, the evaluations for the effect of herbal products or oriental medicines on the pharmacokinetics of a combination drug have been popularly conducted [47,48,49].

In the case of H9, relative percentages of several volatile compounds in H9 were reported, but the identified compounds may not directly correlate with the active constituents responsible for the pharmacological activities of H9 [28,32]. In the preliminary study, the pharmacokinetic profiles of coumarin, isoeugenol, and curcumin were recorded as the high rank of relative percentages in H9 [28,32] were examined (Table S6 and Figures S1–S3). Approximately 0.08% of coumarin was detected in H9, but curcumin and isoeugenol were not detected in H9 (Figure S1). In the pharmacokinetic study of H9, coumarin, isoeugenol, and curcumin were not detected in mouse-plasma samples after oral administration of 500 mg/kg H9 (Table S6 and Figures S1–S3). In addition, coumarin, isoeugenol, and curcumin were also not detected in mouse-plasma samples after oral administration of 500 mg/kg H9 with intravenous administration of 1 mg/kg trastuzumab (Table S6 and Figures S1–S3). In light of the previous reports about anticancer activity of H9 on its own, as well as its synergistic effects with trastuzumab, the emphasis of this study is placed on examining how H9 affects the pharmacokinetic properties of trastuzumab in their combinations.

To address the effect of H9 on trastuzumab pharmacokinetics, ELISA has several advantages (i.e., simple procedure, high specificity, high sensitivity, high efficiency, generally safe and eco-friendly, and cost-effective) compared to other analytical procedures [50,51,52]. As animal models, mice, one of the commonly chosen rodent models, were used because mice have gained increasing popularity in the field of pharmacokinetics. In genetic similarities to humans, mice have approximately 97.5% of their working DNA in common with humans [53]. Mouse models are valuable for developing a wider range of disease models that better reflect human disease states and for animal scale-up studies. Also, they typically require a smaller amount of test compounds compared to rat models [54,55,56,57].

Anticancer activities of H9 against lung and breast cancer mouse models have been reported in both mono- and combination-dosage regimens. The mono treatment of H9 was administrating 400 mg/kg of oral H9 daily for 24 days (for breast cancer model) or 36 days (for lung cancer model). A combination treatment was conducted by co-administrating 400 mg/kg of oral H9 daily with either pemetrexed or trastuzumab for 36 days or 29 days, respectively [31,32]. Based on previous reports, in this study 500 mg/kg of H9 was adopted and its effect on the pharmacokinetics of trastuzumab was examined. After intravenous administration of trastuzumab (1 mg/kg and 10 mg/kg), trastuzumab showed dose-dependent pharmacokinetic properties in mice. There was a 10-fold difference in the AUC values between 1 and 10 mg/kg doses, and other pharmacokinetic parameters were comparable between the two groups.

As trastuzumab was intravenously administered, the absorption phase of trastuzumab was skipped, as it immediately entered systemic circulation. Thus, the therapeutic value of trastuzumab was determined by the disposition (distribution and elimination) [58]. Several studies have already been reported that the pharmacokinetics of trastuzumab are characterized by a low volume of distribution, a slow systemic clearance and a long half-life [59,60].

Regarding distribution, it has been reported that the volume of distribution of trastuzumab is low [61]. Generally, the diffusion of systemically administered monoclonal antibodies is limited in tissues including tumors [59,61]. Multiple factors are involved in low and heterogeneous distribution such as their structure (high molecular weight, affinity for membrane antigens that delay diffusion) [61,62,63,64,65,66,67]. In particular, the lack of diffusion is attributed to the interstitium structure in relation to its negative charges, considering the positive charge of trastuzumab favors its distribution in the tumor interstitium when compared with neutral or negatively charged macromolecules [68]. Although trastuzumab was detected in the liver, heart, lung, spleen, kidney, GI, and muscle at 6 h, 24 h, and 72 h, the corresponding concentrations of trastuzumab were lower than those in the plasma, respectively. These results indicated that trastuzumab had a low affinity for tissues, which was supported by the considerably small V_ss_ value of trastuzumab (Table 3), which was slightly larger than the plasma volume of mice (50 mL/kg) [42,43]. Considering that the total body water of a mouse is 725 mL/kg [43], this result indicated that the distribution of trastuzumab was limited within the tissues and largely confined to the plasma space. The T/P ratio of all tissues < 1.0 (Figure 5 and Table S1) also supported the low volume of distribution of trastuzumab. Trastuzumab showed high uptake in the spleen and kidneys, which are organs of the reticuloendothelial system (RES) and detect external substances based on sufficient blood flow and metabolism [69].

Regarding elimination, the clearance of trastuzumab did not appear to occur by excretion and liver metabolism as with conventional drugs, although the elimination pathway of trastuzumab is unknown [59]. Trastuzumab levels could be regulated in the vascular compartment by endothelial cells via FcRn receptors, which have been expressed in the endothelial cells, monocytes, and epithelial cells of various human tissues. FcRn receptors are known to possess two main functions: the transport of IgG (particularly the transfer of IgG across the maternofetal barrier) known as transcytosis and the control of IgG catabolism [70], and this could explain the long half-life of trastuzumab [71,72]. The CL of trastuzumab was slow, and the terminal half-life and MRT were long (Table 3), suggesting that trastuzumab was slowly eliminated from the body and remained in circulation for an extended period. The intact trastuzumab was not detected in urine over 24 h (Ae_24 h_) (our unpublished data), suggesting that the trastuzumab was not distributed to the kidneys for elimination or was metabolized or degraded and eliminated from the body before it could be excreted in the urine. This means that trastuzumab was highly metabolized by proteolysis in the kidney and then excreted as metabolites [13,73]. In this study, metabolites of trastuzumab were not identified, so further investigation is required to evaluate the metabolic profile of trastuzumab.

It has been reported that the continuous use of trastuzumab can lead to resistance [74]. However, the anti-tumor activity of the trastuzumab and H9 combination against breast cancer in a mouse model was improved after its weekly intravenous administration [28]. As the systemic exposure (represented by AUC) and/or local tissue concentration of a drug can drive its efficacy and toxicity [39,75], the effect of H9 on trastuzumab pharmacokinetics has been investigated.

Regarding the combination of H9 with trastuzumab, single and 2-week daily administration of oral H9 (500 mg/kg daily) did not cause any change of trastuzumab pharmacokinetics in mice. This result indicated that single and 2-week treatment of H9 did not affect the pharmacokinetics of trastuzumab. The CL values of trastuzumab for TM mice with and without H9 were low, and the T/P ratios were less than one in the tissue-distribution study. Moreover, in the H9+TM mice, the AUC_0–72h_ (μg h/g tissue) and T/P ratio patterns (Figure 5 and Table 6) were comparable to those in the trastuzumab-only group. This result indicates that H9 did not affect the distribution of trastuzumab in extravascular sites. In particular, the operational multiple dosing half-life is a key to defining drug accumulation in patients [76], and the rate of accumulation is mainly determined by the biological half-life [77]. The terminal half-lives of trastuzumab were comparable between the TM1 and 2-week H9+TM1 groups (Table 4).

In the multiple-combination dosage regimen of trastuzumab and H9 showing synergistic anti-tumor activity [32] (i.e., intravenously administered 1 mg/kg of trastuzumab twice weekly for 3 weeks and 500 mg/kg of oral H9 daily for 2 weeks), no change in the pharmacokinetic parameters of trastuzumab were observed between the multiple trastuzumab and multiple H9+trastuzumab groups (Table 5). Between the multiple TM1 and multiple H9+TM1 groups, there was no significant difference in the terminal half-life of trastuzumab (Table 5), suggesting multiple oral administrations of H9 did not change the accumulation patterns of trastuzumab in single or multiple doses. In other words, multiple treatment of H9 did not affect trastuzumab pharmacokinetics, including distribution, elimination and accumulation.

This is the first attempt to assess the pharmacokinetic change of trastuzumab by H9, and it confirms the lack of any interference of H9 on systemic and local tissues exposures of trastuzumab. In general, the changes of systemic and local tissue exposure(s) of a drug can impact the efficacy and side effects in combination therapies [78,79,80]. For example, an increase of systemic exposure of etoposide to ketoconazole enhances the anti-cancer effect in etoposide–ketoconazole combination [81], while an increase of systemic exposure of 5-fluorouracil to leucovorin leads to more frequent undesired side effects in 5-fluorouracil–leucovorin combination [82]. An increase of liver distribution of metformin by cimetidine improved glucose-lowering activity in the metformin–cimetidine combination, and a decrease of liver distribution of pravastatin by paroxetine is closely linked to a reduction in the lipid-lowering activity of the pravastatin–paroxetine combination [83]. Considering these examples and interpreting the relationship between exposure and response to changes in combination therapies, our findings suggest that H9 does not influence the systemic and local tissue exposures of trastuzumab in their single and multiple periods of combinations. Consequently, this result supports the absence of any additional toxicity when combining trastuzumab with H9, compared to mono treatment with either trastuzumab or H9.

## 4. Materials and Methods

### 4.1. Materials and Reagents

Trastuzumab (Herceptin^®^; Roche, Basel, Switzerland) and H9 (Han Poong Pharmaceutical Co., Ltd., Seoul, Republic of Korea) were provided by Prof. Seung Hyun Jung (College of Korean Medicine, Dongguk University, Gyeongju, Republic of Korea). A validated ELISA kit was purchased from Eagle Biosciences, Inc. (Nashua, NH, USA). The composition of ELISA was as follows: microtiter plate precoated with ligand antigen (recombinant human HER2/ErbB2/CD340); 3, 1, 0.3, 0.1, and 0 µg/mL of calibration standards for trastuzumab analysis; assay buffer including proteins with less than 0.1% NaN_3_; horse radish peroxidase-conjugated antihuman IgG1 Fc-specific monoclonal antibody (Clone 1B5); TMB substrate solution; TMB stop solution; and washing buffer.

### 4.2. Calibration Standards and QCs

The calibration standards of trastuzumab at 3, 1, 0.3, 0.1, and 0 µg/mL were provided with the ELISA kit, which was used for QCs (3, 1, 0.3 and 0.1 µg/mL) for the validation process.

### 4.3. Sandwich ELISA Procedure and Assay Validation

The trastuzumab concentrations in the biological samples of mice were determined using the validated ELISA kit according to the manufacturers’ instructions. Briefly, 100 μL of assay buffer was added into each well of a 96-well microtiter plate, and 10 μL of each ready-to-use calibrator, QC, and sample was transferred to each well. The plate was covered with adhesive film and incubated at room temperature for 30 min. After removing the adhesive film, the incubated solutions were discarded. Each well was washed by adding 300 μL of the diluted washing buffer at room temperature for 5 min, and the remaining washing buffer was discarded. This washing step was repeated three times. After washing the plate, 100 μL of HRP-conjugated antihuman IgG1 Fc-specific mouse monoclonal antibody (Clone 1B5) as a secondary antibody was added to each well, and the plate was covered with adhesive film and incubated for 60 min at room temperature. Each well in the plate was washed by adding 300 μL of the diluted washing buffer three times. After completing the washing step, 100 μL of the TMB substrate solution was added into each well and incubated for 10 min at room temperature in the dark. To stop the substrate reaction, 100 μL of the stop solution was added into each well, and this mixture in the plate was gently shaken. When the color of the mixture in the plate changed from blue to yellow, the absorbance was measured at 450 nm within 15 min of adding of the stock solution by gently shaking the plate using an ELISA reader (SpectraMax^®^ M3 Multi-mode microplate reader; Molecular devices, San Jose, CA, USA). The standard curve was constructed by plotting the optical density at 450 nm for each standard sample on the vertical linear *y*-axis versus the corresponding concentration of trastuzumab on the horizontal logarithmic *x*-axis.

The accuracy of the ELISA was assessed using QCs (including low, medium and high concentrations, 3, 1, 0.3, and 0.1 µg/mL), and relative errors (%RE = [measured concentration − nominal concentration]/nominal concentration × 100) were calculated. The coefficient of variation values (CV%) of intra-day precision were assessed on the same day and inter-day precision was assessed on four independent days. The nominal concentrations on the standard curve were defined when CV and RE were within 20%, except for the lowest and highest concentrations. The lowest and highest concentrations on the standard curve were defined as the lower limit of quantification (LLOQ) and upper limit of quantification (ULOQ) when CV and RE were within 25%, according to the US FDA bioanalysis method validation guidance for industry [28].

### 4.4. Dilution Effect

The dilution effect of trastuzumab was assessed in mouse-plasma and -tissue samples. A stock solution of trastuzumab (150 mg/mL) was prepared in PBS and serially diluted with assay buffer. The standards of plasma and tissues were spiked with trastuzumab to a final concentration of 1 and 0.1 μg/mL, respectively (n = 5 for each dilution). In plasma, the acceptable dilution ratio was established by testing blank mouse plasma diluted at 1:40, 1:20, 1:10 and 1:2. The dilution ratio of trastuzumab in mouse tissue samples was also evaluated using blank mouse-tissue homogenates (i.e., GI, lung, liver, spleen, muscle, heart and kidney) diluted at 1:40, 1:20, and 1:10 (for all tissues), and 1:2 (for GI). Other analytical procedures of ELISA were the same as those described above in the validation of the ELISA kit.

### 4.5. Stability of Trastuzumab in Biological Samples

The stability of trastuzumab in mouse plasma was assessed. After keeping mouse plasma with 100 and 1 μg/mL of trastuzumab at −80 °C for 1 day (short-term storage), 7 days (mid-term storage), and 12 days (long-term storage), the concentrations of the remaining trastuzumab in mouse plasma were measured. The measured trastuzumab concentrations after storage were compared to the nominal values in day 0 (sample preparation day) [83]. The samples were analyzed as described above in the validation of the ELISA kit (Eagle Biosciences, Nashua, NH, USA). 

### 4.6. Animals

The protocols for the animal studies were approved by the Institute of Laboratory Animal Resources of Dongguk University_Seoul, Republic of Korea (IACUC-2016-003-1). Male 5-week-old ICR mice were purchased from the Charles River Company Korea (Orient, Seoul, Republic of Korea). Upon arrival, animals were randomized and housed at four per cage under strictly controlled environmental conditions (22–25 °C and 48–52% relative humidity) with a 12 h light/dark cycle at an intensity of 150 to 300 Lux. All mice were provided food and water and were then maintained throughout this study.

### 4.7. Trastuzumab Pharmacokinetics after Its Intravenous Administration (1 or 10 mg/kg) with and without Oral H9 (500 mg/kg) in Mice

Mice were randomly divided into four groups: TM1, H9+TM1, TM10 and H9+TM10. On the experimental day, 1 mg (5 mL)/kg of trastuzumab was intravenously administered via the tail vein to the TM1 and H9+TM1 groups, and 10 mg (5 mL)/kg of trastuzumab was intravenously administered via the tail vein in TM10 and H9+TM10 groups. Simultaneously, a dose of 500 mg (10 mL)/kg of H9 was orally administered to the H9+TM1 and H9+TM10 groups, whereas distilled water as a vehicle was orally administered to the TM1 and TM10 groups (Figure 1a). Blood sampling by heart puncture followed the recommended guidelines and the approved protocols of a blood sampling volume of 0.26 mL/35 g body weight (BW) at each time point, and a total blood sampling volume of 2.76 mL/35 g BW [40,84,85,86]. A 31-gauge needle was used to minimize damage to cardiac and pericardial tissues along the needle track and to keep mice alive for several blood collections. Blood samples (approximately 120 µL) were collected via heart puncture using a heparinized insulin syringe at 0, 0.017, 0.5, 6, 12, 24, 48, 72, 96, 168, 240, or 336 h after the trastuzumab injection. Blood samples were immediately centrifuged at 12,000 rpm and 4 °C for 10 min, and 50 µL of plasma was collected and stored at −20 °C for ELISA analysis of trastuzumab. The plasma samples obtained until 72 h in the TM1 and H9+TM1 groups were diluted 20-fold, and those obtained until 72 h in TM10 and H9+TM10 groups were diluted 20- or 40-fold, before conducting the ELISA analysis. 

### 4.8. Trastuzumab Pharmacokinetics after Its Intravenous Administration (1 mg/kg) with and without 2-Week Treatment of Oral H9 (500 mg/kg) in Mice

Mice were randomly divided into two groups: TM1 and 2-week H9+TM1. According to the dosage regimen of H9, to show the in vivo anti-cancer effect of H9 [28], a daily dose of 500 mg (10 mL)/kg of H9 was orally administered for 2 weeks in the 2-week H9+TM1 group, whereas the distilled water as a vehicle was orally administered following the same schedule to the TM1 group (Figure 1b). On the experimental day, 1 mg (5 mL)/kg of trastuzumab was intravenously administered via the tail vein to both groups. Other procedures for the pharmacokinetic study were the same as those described in the pharmacokinetic study of trastuzumab. In the ELISA analysis, the plasma samples obtained until 72 h were diluted 20-fold.

### 4.9. Trastuzumab Pharmacokinetics after Multiple Intravenous Administration of Trastuzumab (1 mg/kg) with and without Oral H9 (500 mg/kg) in Mice

Mice were randomly divided into two groups: multiple TM1 and multiple H9+TM1. According to the dosage regimen of the trastuzumab and H9 combination showing their synergistic effect in vivo [28], multiple treatments of intravenous trastuzumab with and without oral H9 were conducted (Figure 1c). From 3 weeks before the experiment, 1 mg (5 mL)/kg of trastuzumab was intravenously administered via the tail vein twice weekly for 3 weeks in the multiple TM1 and multiple H9+TM1 groups. From 2 weeks before the experiment, 500 mg (10 mL)/kg of H9 was orally administered daily in the multiple H9+TM1 group, whereas distilled water was orally administered daily in the multiple TM1 group, respectively. On the experimental day, the same dosage regimen of trastuzumab and H9 was conducted in the multiple TM1 and multiple H9+TM1 groups, respectively. Other procedures for the pharmacokinetic study were the same as those described in the pharmacokinetic study of trastuzumab. In the ELISA analysis, the plasma samples obtained until 72 h were diluted 20-fold.

### 4.10. Tissue Distribution of Trastuzumab after Its Intravenous Administration (10 mg/kg) with and without Oral H9 (500 mg/kg) in Mice

Mice were randomly divided into two groups: TM10 and H9+TM10 mice. On the experimental day, 10 mg (5 mL)/kg of trastuzumab was intravenously administered via the tail vein in the TM10 and H9+TM10 groups. H9 (500 mg (10 mL)/kg) was orally administered only to the H9+TM10 group, whereas distilled water was orally administered to the TM10 group. At 6, 24, and 72 h after drug administration, whole blood was collected via the cardiac puncture and 0.9% injectable NaCl solution was perfused via the portal vein. The blood sample was centrifuged, and a 0.1-mL plasma sample was collected. Following complete systemic perfusion with the 0.9% injectable NaCl solution, several tissues such as GI, lung, liver, spleen, muscle, heart, and kidney were excised, washed with the 0.9% injectable NaCl solution, and blotted dry with tissue paper. After weighing each tissue, the samples were homogenized in a 4-fold volume of ELISA standard dilution buffer using a tissue homogenizer. The homogenized samples were centrifuged at 12,000 rpm and 4 °C for 10 min, and then the supernatants were collected. All samples were stored at −80 °C until the ELISA analysis. In the ELISA analysis, different dilution factors were used depending on the tissues: 2-fold dilution for the GI, and 10, 20, and 40-fold dilution for the GI, lung, liver, spleen, muscle, heart, and kidneys.

### 4.11. Pharmacokinetic Analysis

Pharmacokinetic parameters of trastuzumab were determined using two-compartmental analysis [87] using PK solver (version 2.1; Scientific) as follows: AUC_0–336h_ or AUC_0–∞_, terminal half-life, CL, MRT and V_ss_.

A two-compartment model was used to establish a pharmacokinetic model of trastuzumab. The following differential equations were used:

$$\frac{dX_{c}}{dt\;}={-k}_{12}\cdot X_{c}-k_{e}\cdot X_{c}+k_{21}\cdot X_{p}$$

$$\frac{dX_{p}}{dt\;}={-k}_{12}\cdot X_{c}-k_{21}\cdot X_{p}$$

In the above, the *k*_12_ (1/h) and *k*_21_ (1/h) are the apparent first-order intercompartmental distribution constants (or transfer rate constants); *k*_e_ (1/h) is the apparent first-order elimination rate constant from the central compartment; and *X*_c_ (mg) and *X*_p_ (mg) are the drug amount in the central and peripheral compartments, respectively.

### 4.12. Statistical Analysis

A *p* value < 0.05 was deemed statistically significant using a student *t*-test between the two means for unpaired data or a Statistical Package for the Social Sciences (SPSS; version 27) analysis of variance among three groups. All data are expressed as mean and SD.

## 5. Conclusions

The intravenous administration of trastuzumab (1 or 10 mg/kg) with and without oral H9 (500 mg/kg) revealed that H9 did not affect the pharmacokinetic properties of trastuzumab. In addition, trastuzumab showed linear pharmacokinetic patterns after its intravenous administration in the range from 1 mg/kg to 10 mg/kg. Moreover, single or multiple (twice weekly for 3 weeks) intravenous administrations of trastuzumab (1 mg/kg) with and without oral H9 (500 mg/kg) demonstrated that accumulation of trastuzumab was not observed in multiple administrations of trastuzumab and multiple (daily for 2 weeks) administrations of oral H9 (500 mg/kg) did not affect the pharmacokinetic properties, including the accumulation patterns of trastuzumab. In exposures of trastuzumab, H9 does not influence the systemic and local tissue exposures of trastuzumab in single and multiple periods of their combinations, supporting the absence of any additional toxicity when combining trastuzumab with H9, compared to mono-treatment with either trastuzumab or H9. Furthermore, it can be suggested that the synergism observed in trastuzumab and H9 combinations may arise from the alternation in the trastuzumab signaling pathways initiated or affected by H9 after trastuzumab reaches tumor cells, or from the inherent pharmacological activities of H9 itself. Although further translational studies are necessary, these results provide helpful information to interpret the pharmacokinetic characteristics of trastuzumab in single, as well as combination, therapy with H9 and suggest the possibility of stable breast cancer treatment through the synergistic effect of the co-treatment of trastuzumab with H9.

## Figures and Tables

**Table 1:** Required dilution factors for measurement of trastuzumab concentrations in plasma and various mouse tissues.

Dilution Factors	1:40		1:20		1:10		1:2	
Nominal Concentration (μg/mL)	0.100	1.00	0.100	1.00	0.100	1.00	0.100	1.00
Plasma								
Measured concentration (μg/mL)	0.0992	1.05	0.104	1.036	0.100	0.972	0.103	0.970
SD (μg/mL)	0.00273	0.0938	0.00547	0.0751	0.00718	0.0615	0.00789	0.519
Accuracy (bias %)	−0.81	4.87	3.96	3.59	−0.155	−2.78	3.22	−2.99
Precision (CV %)	2.75	8.94	5.26	7.25	7.19	6.32	7.64	5.35
GI								
Measured concentration (μg/mL)	0.100	1.05	0.0962	1.02	0.101	1.03	0.104	1.03
SD (μg/mL)	0.00714	0.0522	0.0115	0.0833	0.00730	0.0673	0.00536	0.0542
Accuracy (bias %)	0.0211	5.09	−3.79	1.80	0.570	2.59	3.51	2.68
Precision (CV %)	7.14	4.96	12.0	8.18	7.26	6.56	5.18	5.28
Lung								
Measured concentration (μg/mL)	0.0993	0.968	0.104	1.02	0.0952	1.02		
SD (μg/mL)	0.00869	0.0615	0.00632	0.0798	0.00566	0.0516		
Accuracy (bias %)	−0.720	−3.19	3.65	2.31	−4.78	1.78		
Precision (CV %)	8.76	6.35	6.10	7.80	5.94	5.07		
Liver								
Measured concentration (μg/mL)	0.0974	1.03	0.101	1.041	0.0970	1.01		
SD (μg/mL)	0.00744	0.0872	0.00724	0.0594	0.00359	0.0810		
Accuracy (bias %)	−2.56	3.30	0.924	4.10	−3.04	0.801		
Precision (CV %)	7.63	8.44	7.18	5.71	3.71	8.04		
Spleen								
Measured concentration (μg/mL)	0.103	1.00	0.103	0.972	0.109	1.04		
SD (μg/mL)	0.00622	0.0519	0.00655	0.0801	0.00541	0.0480		
Accuracy (bias %)	2.96	0.156	2.85	−2.79	9.23	3.58		
Precision (CV %)	6.04	5.18	6.37	8.24	4.95	4.63		
Muscle								
Measured concentration (μg/mL)	0.0987	0.100	0.105	1.04	0.106	1.05		
SD (μg/mL)	0.00606	0.0711	0.00354	0.0703	0.00646	0.0715		
Accuracy (bias %)	−1.28	−0.0202	4.65	3.94	5.69	5.31		
Precision (CV %)	6.14	7.11	3.38	6.77	6.11	6.79		
Heart								
Measured concentration (μg/mL)	0.100	1.02	0.100	1.01	0.107	1.04		
SD (μg/mL)	0.00463	0.0791	0.00602	0.0986	0.0106	0.0599		
Accuracy (bias %)	0.206	2.24	0.167	0.845	6.88	3.71		
Precision (CV %)	4.62	7.74	6.01	9.78	9.96	5.78		
Kidney								
Measured concentration (μg/mL)	0.101	1.03	0.103	1.03	0.100	1.04		
SD (μg/mL)	0.00644	0.0774	0.00623	0.0521	0.00869	0.0639		
Accuracy (bias %)	1.06	3.03	2.98	2.78	−0.230	3.63		
Precision (CV %)	6.37	7.51	6.05	5.07	8.71	6.17		

**Table 2:** Accuracy, precision, and inter- and intra-day measurements of trastuzumab concentrations in plasma and various mouse tissues.

Nominal Concentration (μg/mL)	Measured Concentration (μg/mL)				Accuracy (Bias %)		Precision (CV %)	
	Inter-Day		Intra-Day		Inter-Day	Intra-Day	Inter-Day	Intra-Day
	Mean	SD	Mean	SD				
Plasma								
0.1	0.101	0.00631	0.0997	0.00675	1.22	−0.274	6.23	6.77
0.3	0.308	0.0160	0.310	0.0224	2.50	3.44	5.19	7.23
1	1.01	0.0639	0.977	0.0526	1.01	−2.30	6.32	5.38
3	3.01	0.141	3.03	0.156	0.339	0.875	4.68	5.15
GI								
0.1	0.0978	0.00773	0.0996	0.00634	−2.18	−0.445	7.91	6.37
0.3	0.305	0.0154	0.308	0.0173	1.54	2.73	5.07	5.62
1	1.01	0.0695	1.04	0.0779	1.47	3.58	6.85	7.52
3	3.09	0.185	3.19	0.0642	3.06	6.31	6.00	2.01
Lung								
0.1	0.101	0.00641	0.102	0.00402	1.02	2.17	6.34	3.94
0.3	0.303	0.0171	0.309	0.0272	0.979	3.05	5.66	8.80
1	1.01	0.0637	0.999	0.0588	0.935	−0.113	6.31	5.89
3	3.07	0.111	3.08	0.145	2.34	2.59	3.63	4.72
Liver								
0.1	0.0979	0.00499	0.0979	0.00347	−2.10	−2.08	5.10	3.54
0.3	0.303	0.0117	0.305	0.0129	1.12	1.64	3.85	4.25
1	1.01	0.0631	1.02	0.0452	1.29	2.11	6.23	4.43
3	3.01	0.120	3.12	0.0864	0.207	3.84	3.99	2.77
Spleen								
0.1	0.102	0.00583	0.104	0.00453	1.74	4.12	5.73	4.35
0.3	0.299	0.0163	0.300	0.0196	−0.260	0.151	5.44	6.51
1	0.983	0.0711	1.01	0.0609	−1.66	0.878	7.23	6.04
3	2.97	0.138	2.93	0.179	−1.09	−2.20	4.65	6.09
Muscle								
0.1	0.103	0.00562	0.0993	0.00442	3.07	−0.701	5.46	4.45
0.3	0.311	0.0139	0.305	0.0130	3.54	1.82	4.49	4.26
1	1.02	0.0797	0.992	0.0775	1.69	−0.762	7.84	7.80
3	3.02	0.122	3.10	0.0858	0.759	3.18	4.03	2.77
Heart								
0.1	0.100	0.00584	0.0985	0.00565	−0.0134	−1.49	5.84	5.74
0.3	0.302	0.0150	0.295	0.0127	0.682	−1.79	4.98	4.31
1	1.02	0.0819	1.03	0.0712	2.13	3.06	8.02	6.91
3	3.01	0.125	3.13	0.0760	0.476	4.21	4.13	2.43
Kidney								
0.1	0.100	0.00688	0.104	0.00770	0.190	3.96	6.86	7.40
0.3	0.312	0.0140	0.310	0.0182	4.16	3.40	4.47	5.87
1	1.01	0.0492	0.985	0.0583	1.03	−1.45	4.87	5.91
3	2.99	0.120	2.93	0.124	−0.376	−2.19	4.02	4.23

**Table 3:** Mean (±SD) pharmacokinetic parameters of trastuzumab after its intravenous administration at a dose of 1 mg/kg with and without 500 mg/kg of oral H9 to TM1 and H9+TM1 mice. The corresponding values after intravenous administration of trastuzumab at a dose of 10 mg/kg with and without 500 mg/kg of oral H9 to TM10 and H9+TM10 mice.

Parameters	H9+TM1	TM1	H9+TM10	TM10
	(*n* = 6)	(*n* = 7)	(*n* = 6)	(*n* = 6)
Body weight (g)	38.2 ± 1.17	37.7 ± 0.756	36.8 ± 1.83	36.2 ± 1.17
AUC_0–336 h_ (μg h/mL)	656 ± 115	715 ± 109	6847 ± 713	6885 ± 1166
AUC_0–∞_ (μg h/mL)	873 ± 117	983 ± 105	8245 ± 941	8835 ± 1936
Terminal half-life (h)	139 ± 17.3	164 ± 32.9	134 ± 14.6	156 ± 32.6
CL (mL/h/kg)	1.16 ± 0.159	1.03 ± 0.111	1.23 ± 0.157	1.18 ± 0.284
MRT (h)	193 ± 24.6	227 ± 44.8	185 ± 21.5	215 ± 44.8
V_ss_ (mL/kg)	223 ± 23.0	231 ± 39.7	226 ± 23.6	247 ± 38.9

**Table 4:** Mean (±SD) pharmacokinetic parameters of trastuzumab after its intravenous administration at a dose of 1 mg/kg with and without 500 mg/kg of oral H9 to TM1 and 2-week H9+TM1 mice. H9 (500 mg/kg) was orally administered daily for 2 weeks before trastuzumab administration.

Parameters	2-Week H9+TM1	TM1
	(*n* = 6)	(*n* = 6)
Body weight (g)	45.2 ± 3.19	44.7 ± 2.88
AUC_0–336 h_ (μg h/mL)	758 ± 154	795 ± 208
AUC_0–∞_ (μg h/mL)	1012 ± 141	1095 ± 221
Terminal half-life (h)	150 ± 24.4	163 ± 37.3
CL (mL/h/kg)	1.00 ± 0.136	0.947 ± 0.203
MRT (h)	208 ± 35.2	226 ± 51.3
V_ss_ (mL/kg)	208 ± 40.2	213 ± 58.0

**Table 5:** Mean (±SD) pharmacokinetic parameters of trastuzumab after its intravenous administration at a dose of 1 mg/kg with and without 500 mg/kg of oral H9 in multiple TM1 and multiple H9+TM1 mice, respectively. Trastuzumab (1 mg/kg) was intravenously administered twice weekly for 3 weeks, and H9 (500 mg/kg) was orally administered once daily for 2 weeks in multiple H9+TM1 mice, whereas multiple TM1 mice were pretreated with the same dose and frequency of trastuzumab without 500 mg/kg of oral H9, respectively.

Parameters	Multiple H9+TM1	Multiple TM1
	(*n* = 5)	(*n* = 6)
Body weight (g)	47.0 ± 1.58	47.3 ± 2.80
AUC_0–336 h_ (μg h/mL)	897 ± 101	770 ± 214
AUC_0–∞_ (μg h/mL)	1156 ± 138	1073 ± 187
Terminal half-life (h)	141 ± 40.3	159 ± 35.1
CL (mL/h/kg)	0.876 ± 0.110	0.963 ± 0.213
MRT (h)	199 ± 54.2	219 ± 45.2
V_ss_ (mL/kg)	170 ± 32.3	208 ± 45.9

**Table 6:** Mean (±SD) AUC_0–72 h_ (μg h/g tissue) of trastuzumab in various tissues after intravenous administration of trastuzumab at a dose of 10 mg/kg with and without 500 mg/kg of oral H9 to mice, respectively.

Tissue	H9+TM10	TM10
	(*n* = 5)	(*n* = 5)
Liver	379 ± 67.0	381 ± 88.6
Heart	366 ± 40.5	315 ± 90.1
Lung	441 ± 87.4	451 ± 34.6
Spleen	545 ± 51.7	623 ± 158
Kidney	279 ± 53.1	260 ± 53.5
GI	90.8 ± 18.5	80.9 ± 18.4
Muscle	47.2 ± 13.5	54.6 ± 20.8

## Data Availability

The data are contained within this article.

## Supplementary Materials

The following supporting information can be downloaded at https://www.mdpi.com/article/10.3390/ijms242316677/s1.
