# Supplementary material for: No Interference of H9 Extract on Trastuzumab Pharmacokinetics in Their Combinations
Source: Int J Mol Sci. 2023 Nov 23;24(23):16677. doi: 10.3390/ijms242316677 (PMC10706748; doi:10.3390/ijms242316677)
Supplement: Supplementary file 1 [file ijms-24-16677-s001.zip › ijms-2656246-supplementary.pdf]

Table S1. Mean (± SD) concentrations (μg/mL for plasma and μg/g tissue) of trastuzumab in plasma and tissues at 6, 24, and 72 h after intravenous administration of trastuzumab at a dose of 10 mg/kg with and without 500 mg/kg of oral H9 to mice, respectively.

Tissue	6 h (*n* = 5)		24 h (*n* = 5)		72 h (*n* = 5)	
	H9+TM10	TM10	H9+TM10	TM10	H9+TM10	TM10
Plasma	60.3 ± 9.03	56.9 ± 7.66	32.7 ± 2.32	40.2 ± 12.1	20.9 ± 4.30	20.7 ± 4.61
Liver	9.86 ± 1.37	9.86 ± 1.79	6.14 ± 1.56	5.81 ± 2.32	3.06 ± 1.51	3.53 ± 1.43
	(0.164 ± 0.00976)	(0.179 ± 0.0554)[a]	(0.188 ± 0.0477)	(0.147 ± 0.0444)	(0.151 ± 0.0840)	(0.177 ± 0.0828)
Heart	10.3 ± 1.62	9.23 ± 1.84	5.80 ± 1.14	4.94 ± 1.38	2.80 ± 1.00	2.38 ± 1.71
	(0.172 ± 0.0269)	(0.163 ± 0.0291)	(0.178 ± 0.0349)	(0.129 ± 0.0361)	(0.140 ± 0.0614)	(0.124 ± 0.103)
Lung	9.83 ± 1.59	10.6 ± 1.86	7.10 ± 1.66	7.58 ± 2.53	4.08 ± 1.22	3.88 ± 1.08
	(0.163 ± 0.0174)	(0.187 ± 0.0298)	(0.218 ± 0.0509)	(0.197 ± 0.0597)	(0.208 ± 0.0820)	(0.188 ± 0.0381)
Spleen	11.8 ± 1.82	12.6 ± 1.86	8.96 ± 1.30	11.8 ± 5.39	4.98 ± 1.23	4.76 ± 1.13
	(0.201 ± 0.0461)	(0.226 ± 0.0487)	(0.274 ± 0.0399)	(0.304 ± 0.115)	(0.250 ± 0.0807)	(0.242 ± 0.0864)
Kidney	9.17 ± 0.991	8.39 ± 1.58	4.47 ± 0.971	4.70 ± 1.05	1.74 ± 1.07	1.70 ± 1.01
	(0.153 ± 0.0153)	(0.150 ± 0.0359)	(0.137 ± 0.0297)	(0.116 ± 0.0670)	(0.0869 ± 0.0552)	(0.0842 ± 0.0447)
GI	1.77 ± 0.883	2.14 ± 0.797	1.98 ± 0.864	2.18 ± 0.950	0.323 ± 0.174	0.356 ± 0.185
	(0.0285 ± 0.0108)	(0.0373 ± 0.0115)	(0.0607 ± 0.0265)	(0.0570 ± 0.0228)	(0.0151 ± 0.00693)	(0.0170 ± 0.0080)
Muscle	1.30 ± 0.474	1.38 ± 0.498	1.11 ± 0.693	1.22 ± 0.848	0.155 ± 0.0958	0.243 ± 0.127
	(0.0224 ± 0.0114)	(0.0241 ± 0.00758)	(0.0339 ± 0.0212)	(0.0607 ± 0.0265)	(0.00754 ± 0.00470)	(0.0112 ± 0.00387)

[a] Values in parentheses are mean values of the tissue to plasma (T/P) ratio.

Table S2. The non-compartmental equations.

**Parameters**	**Equations**
$AUC_{0\text{-}336h}$ or $AUC_{0\text{-}\infty}$ (μg h/mL)	Following the trapezoidal rule: $\sum_{0}^{336h} \frac{t_2 - t_1}{2}(C_{t_1} + C_{t_2})$ or $\sum_{0}^{336h} \frac{t_2 - t_1}{2}(C_{t_1} + C_{t_2}) + \frac{C_{last}}{k}$
Terminal half-life (h)	$\frac{ln\ (2)}{k}$
CL (mL/h/kg)	$\frac{Dose}{AUC_{0-\infty}}$
AUMC (μg h^2/mL)	$\int_0^\infty t \cdot C_t dt$
MRT (h)	$\frac{AUMC_{0-\infty}}{AUC_{0-\infty}}$
V_{ss} (mL/kg)	$MRT \cdot CL$

C_t: plasma concentration of a drug at time *t*, *k*: elimination rate constant of teminal phase.

Table S3. Mean (± SD) pharmacokinetic parameters of trastuzumab after its intravenous administration at a dose of 1 mg/kg with and without 500 mg/kg of oral H9 to TM1 and H9+TM1 mice. The corresponding values after intravenous administration of trastuzumab at a dose of 10 mg/kg with and without 500 mg/kg of oral H9 to TM10 and H9+TM10 mice. These parameters were calculated by non-compartment model analysis.

**Parameters**	**H9+TM1 (*n* = 6)**	**TM1 (*n* = 7)**	**H9+TM10 (*n* = 6)**	**TM10 (*n* = 6)**
Body weight (g)	38.2 ± 1.17	37.7 ± 0.756	36.8 ± 1.83	36.2 ± 1.17
$AUC_{0\text{-}336h}$ (μg h/mL)	640 ± 94.0	713 ± 109	6709 ± 806	6804 ± 1179
$AUC_{0\text{-}\infty}$ (μg h/mL)	866 ± 115	980 ± 108	8129 ± 978	8737 ± 1952
Terminal half-life (h)	139 ± 18.5	167 ± 37.0	132 ± 13.2	160 ± 30.0
CL (mL/h/kg)	1.17 ± 0.158	1.03 ± 0.116	1.25 ± 0.166	1.20 ± 0.286
MRT (h)	198 ± 28.6	228 ± 48.5	186 ± 22.3	217 ± 40.5
V_{ss} (mL/kg)	228 ± 18.0	233 ± 40.5	230 ± 29.5	254 ± 43.6

Table S4. Mean (± SD) pharmacokinetic parameters of trastuzumab after its intravenous administration at a dose of 1 mg/kg with and without 500 mg/kg of oral H9 to TM1 and 2-week H9+TM1 mice. H9 (500 mg/kg) was orally administered daily for 2 weeks before trastuzumab administration. These parameters were calculated by non-compartment model analysis.

**Parameters**	**2-week H9+TM1 (*n* = 6)**	**TM1 (*n* = 6)**
Body weight (g)	45.2 ± 3.19	44.7 ± 2.88
$AUC_{0\text{-}336\,h}$ (μg h/mL)	750 ± 147	790 ± 204
$AUC_{0\text{-}\infty}$ (μg h/mL)	1007 ± 142	1093 ± 224
Terminal half-life (h)	148 ± 25.0	165 ± 41.3
CL (mL/h/kg)	1.01 ± 0.141	0.950 ± 0.207
MRT (h)	209 ± 37.3	228 ± 55.3
V_{ss} (mL/kg)	210 ± 40.1	214 ± 57.4

Table S5. Mean (± SD) pharmacokinetic parameters of trastuzumab after its intravenous administration at a dose of 1 mg/kg with and without 500 mg/kg of oral H9 in multiple TM1 and multiple H9+TM1 mice, respectively. Trastuzumab (1 mg/kg) was intravenously administered twice weekly for 3 weeks, and H9 (500 mg/kg) was orally administered once daily for 2 weeks in multiple H9+TM1 mice, whereas multiple TM1 mice were pretreated with the same dose and frequency of trastuzumab without 500 mg/kg of oral H9, respectively. These parameters were calculated by non-compartment model analysis.

Parameters	Multiple H9+TM1 (*n* = 5)	Multiple TM1 (*n* = 6)
Body weight (g)	47.0 ± 1.58	47.3 ± 2.80
AUC_{0-336h} (μg h/mL)	879 ± 87.0	763 ± 207
$AUC_{0-\infty}$ (μg h/mL)	1155 ± 138	1053 ± 173
Terminal half-life (h)	145 ± 37.3	156 ± 21.7
CL (mL/h/kg)	0.877 ± 0.112	0.976 ± 0.200
MRT (h)	206 ± 57.1	214 ± 26.5
V_{ss} (mL/kg)	176 ± 33.2	208 ± 41.3

Table S6. Peak areas and calculated concentrations of coumarin, isoeugenol, and curcumin in plasma samples after oral administration of H9 with (*n*=3) and without (*n*=3) intravenous administration of trastuzumab in mice. Analysis of coumarin, isoeugenol, and curcumin in plasma samples was conducted using LC-MS/MS method described in **Figure S2**. In pharmacokinetic study, 500 mg (10 mL)/kg of H9 was orally administered to H9 and H9+TM1 groups, and 1 mg (5 mL)/kg of trastuzumab was intravenously administered via the tail vein to H9+TM1 group, respectively.

**Sample**	**Coumarin** *m/z* [M+H]+ 147.053→91.200 Peak area	**Isoeugenol** *m/z* [M+H]+ 165.013→137.389 Peak area	**Curcumin** *m/z* [M+H]+ 369.155→176.900 Peak area	**IS** *m/z* [M+H]+ 237.193→194.100 Peak area
H9-1 (10 min)	1.78E+02 (BD)	2.42E+02 (BD)	8.02E+02 (BD)	6.81E+05
H9-1 (30 min)	1.46E+02 (BD)	1.32E+02 (BD)	6.01E+02 (BD)	6.81E+05
H9-1 (60 min)	1.33E+02 (BD)	1.42E+02 (BD)	7.11E+02 (BD)	6.80E+05
H9-1 (120 min)	1.53E+02 (BD)	1.65E+02 (BD)	8.22E+02 (BD)	6.55E+05
H9-1 (240 min)	2.06E+02 (BD)	2.43E+02 (BD)	7.87E+02 (BD)	6.63E+05
H9-1 (360 min)	2.66E+02 (BD)	1.65E+02 (BD)	8.09E+02 (BD)	6.67E+05
H9-2 (10 min)	3.07E+02 (BD)	2.51E+02 (BD)	5.58E+02 (BD)	6.93E+05
H9-2 (30 min)	3.04E+02 (BD)	2.04E+02 (BD)	8.96E+02 (BD)	6.71E+05
H9-2 (60 min)	2.32E+02 (BD)	2.62E+02 (BD)	4.65E+02 (BD)	6.90E+05
H9-2 (120 min)	6.18E+02 (BD)	2.95E+02 (BD)	8.99E+02 (BD)	6.60E+05
H9-2 (240 min)	1.13E+02 (BD)	1.55E+02 (BD)	6.31E+02 (BD)	6.73E+05
H9-2 (360 min)	2.69E+02 (BD)	1.49E+02 (BD)	5.86E+02 (BD)	6.52E+05
H9-3 (10 min)	1.47E+02 (BD)	1.09E+02 (BD)	6.35E+02 (BD)	6.37E+05
H9-3 (30 min)	2.22E+02 (BD)	2.21E+02 (BD)	8.30E+02 (BD)	6.62E+05
H9-3 (60 min)	1.24E+02 (BD)	1.99E+02 (BD)	5.10E+02 (BD)	6.61E+05
H9-3 (120 min)	2.92E+02 (BD)	2.53E+02 (BD)	5.57E+02 (BD)	6.76E+05
H9-3 (240 min)	1.55E+02 (BD)	3.52E+02 (BD)	6.77E+02 (BD)	6.97E+05
H9-3 (360 min)	1.36E+02 (BD)	1.95E+02 (BD)	4.82E+02 (BD)	6.69E+05
H9+TM1-1 (10 min)	1.43E+02 (BD)	2.01E+02 (BD)	5.25E+02 (BD)	6.44E+05
H9+TM1-1 (30 min)	2.65E+02 (BD)	1.46E+02 (BD)	5.10E+02 (BD)	6.67E+05
H9+TM1-1 (60 min)	1.38E+02 (BD)	1.85E+02 (BD)	7.70E+02 (BD)	6.41E+05
H9+TM1-1 (120 min)	2.60E+02 (BD)	2.44E+02 (BD)	6.74E+02 (BD)	6.87E+05
H9+TM1-1 (240 min)	1.89E+02 (BD)	3.12E+02 (BD)	5.27E+02 (BD)	6.69E+05
H9+TM1-1 (360 min)	1.34E+02 (BD)	2.16E+02 (BD)	4.33E+02 (BD)	6.34E+05
H9+TM1-2 (10 min)	2.56E+02 (BD)	1.99E+02 (BD)	6.13E+02 (BD)	6.83E+05
H9+TM1-2 (30 min)	2.46E+02 (BD)	2.06E+02 (BD)	7.45E+02 (BD)	6.64E+05
H9+TM1-2 (60 min)	1.98E+02 (BD)	2.55E+02 (BD)	4.36E+02 (BD)	6.93E+05
H9+TM1-2 (120 min)	2.69E+02 (BD)	2.48E+02 (BD)	8.12E+02 (BD)	6.87E+05
H9+TM1-2 (240 min)	1.73E+02 (BD)	1.73E+02 (BD)	5.73E+02 (BD)	6.49E+05
H9+TM1-2 (360 min)	2.42E+02 (BD)	1.69E+02 (BD)	5.20E+02 (BD)	6.57E+05
H9+TM1-3 (10 min)	1.66E+02 (BD)	2.11E+02 (BD)	5.79E+02 (BD)	6.82E+05
H9+TM1-3 (30 min)	1.56E+02 (BD)	1.55E+02 (BD)	6.69E+02 (BD)	6.81E+05
H9+TM1-3 (60 min)	1.23E+02 (BD)	1.72E+02 (BD)	7.56E+02 (BD)	6.81E+05
H9+TM1-3 (120 min)	1.85E+02 (BD)	1.85E+02 (BD)	7.94E+02 (BD)	6.66E+05
H9+TM1-3 (240 min)	1.91E+02 (BD)	2.23E+02 (BD)	8.16E+02 (BD)	6.67E+05
H9+TM1-3 (360 min)	2.44E+02 (BD)	1.85E+02 (BD)	8.41E+02 (BD)	6.67E+05

Values in parenthesis mean the calculated concentration of each compound in plasma sample. BD indicates that the concentrations in plasma samples are below detection limit.

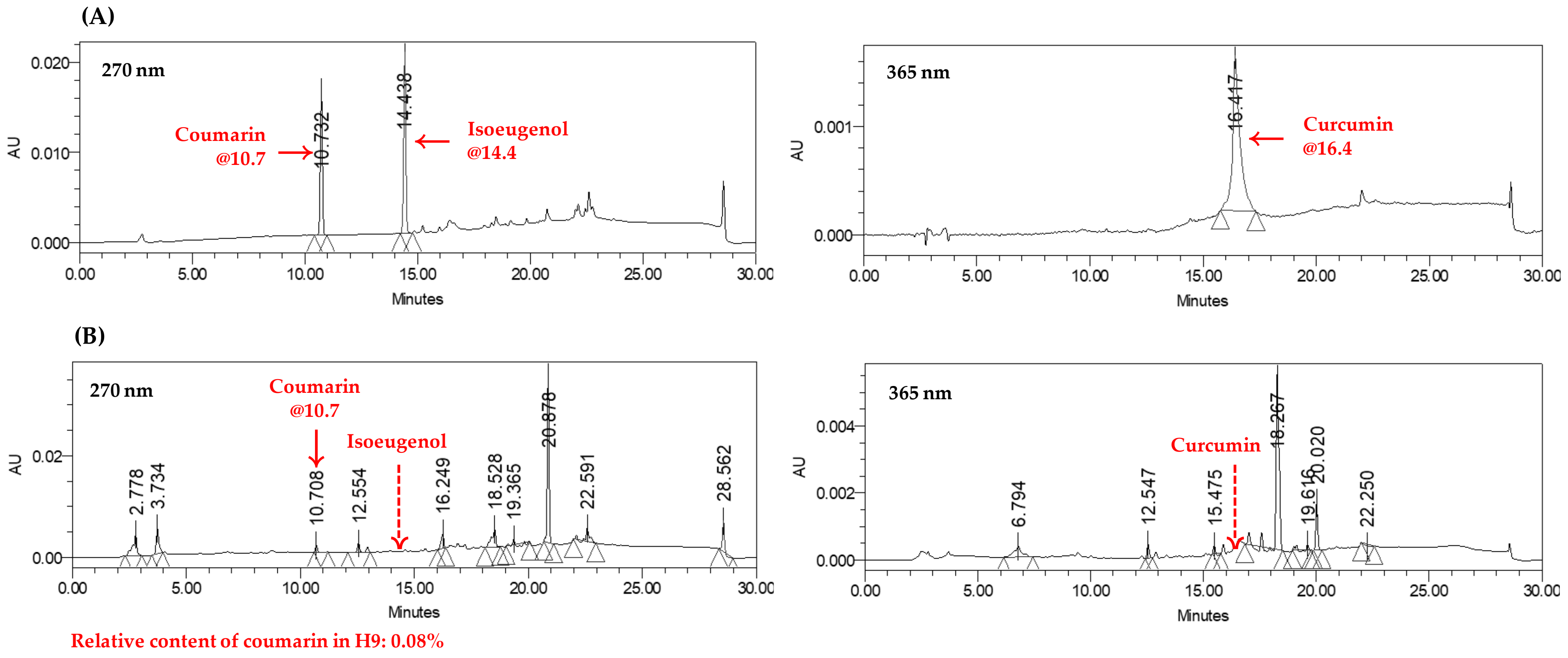

Figure S1. Representative chromatograms of coumarin, isoeugenol, curcumin, and H9 analyzed by HPLC-UV. (A) stock solution of 1 μ g/mL coumarin, 0.002 μ g/mL isoeugenol, and 1 μ g/mL curcumin; (B) stock solution of 100 μ g/mL H9. Analytical HPLC apparatus was consisted of an Allience e2695 pump (Waters, Ireland) and Waters UV/visible detector 2489 (Waters, Ireland). HPLC separations were performed by an Aegispak C_{18}-L column (4.6 × 250 mm, 5 μm) (Young Jin Biochrome Co., Ltd, Republic of Korea) with the following mobile phase condition: 100% distilled water (A) and 100% acetonitrile + 100% methanol (50:50, *v/v*) (B) with a flow rate of 1 mL/min. Gradient elution was performed using the mobile phase at 80:20 (*v/v*) ratio of A:B initially. The ratio was changed to 0:100 (*v/v*) to 18.5 min and was maintained for 25 min. Finally, the ratio was returned to the initial composition at 25.1 min, which was then maintained for 30 min. HPLC method was used for the analysis of coumarin, isoeugenol, and curcumin at 35 °C by using an UV/visible detector at a wavelength of 270 nm for coumarin and isoeugenol, and 365 nm for curcumin, respectively.

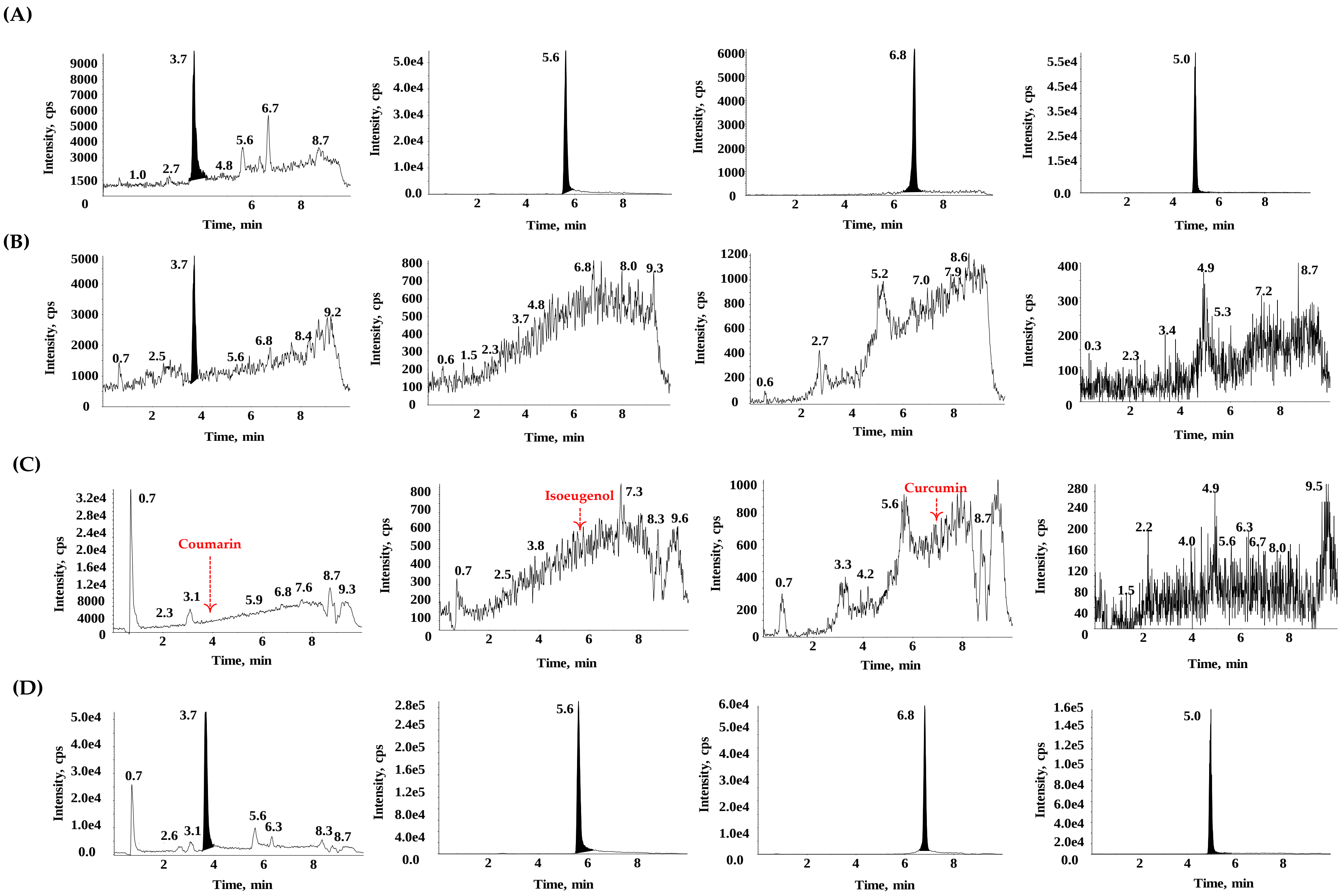
(A)
(B)
(C)
(D)
Intensity, cps
Time, min
Coumarin
Isoeugenol
Curcumin

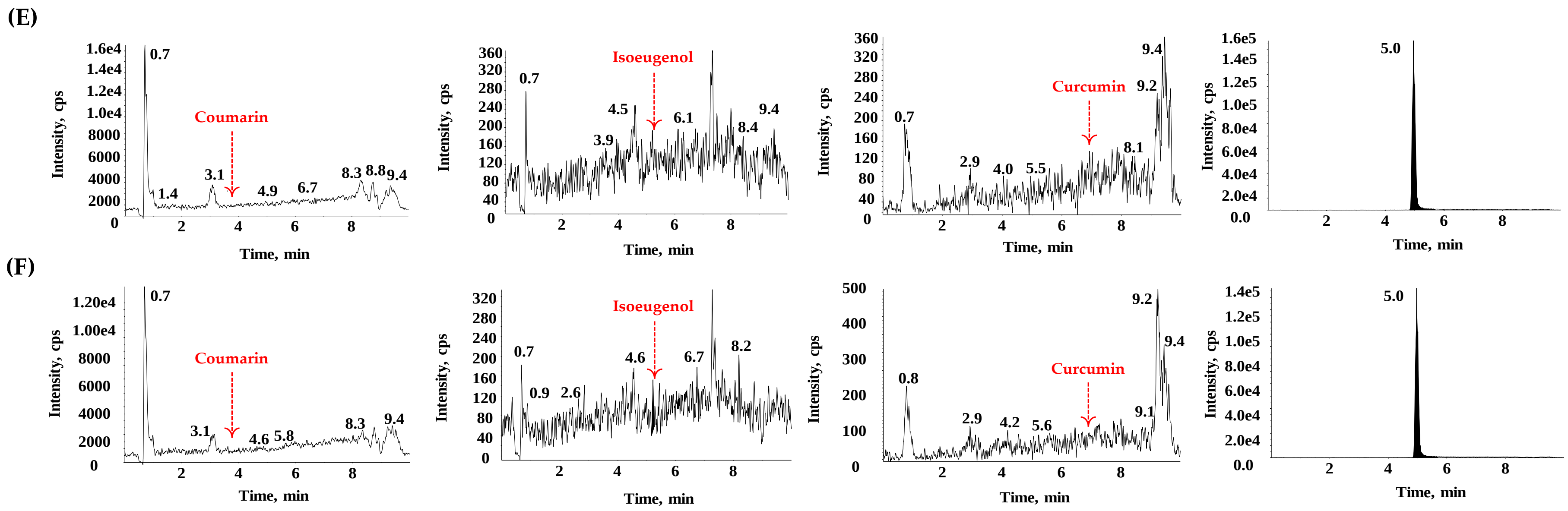

Figure S2. Representative chromatograms of coumarin, isoeugenol, curcumin, and H9 analyzed by LC-MS/MS. (A) stock solution of 0.02 μ g/mL coumarin, 0.01 μ g/mL isoeugenol, 0.02 μ g/mL curcumin, and 0.0025 μ g/mL IS; (B) stock solution of 10 μ g/mL H9; (C) drug-free mouse plasma; (D) plasma standard spiked with 50 μ g/mL coumarin, 25 μ g/mL isoeugenol, and 50 μ g/mL curcumin; (E) plasma sample at 30 min after oral administration of 500 mg/kg H9; (F) plasma sample at 30 min after oral administration of 500 mg/kg H9 and intravenous administration of 1 mg/kg trastuzumab. All analyses were performed using an API 4000 triple quadrupole mass spectrometer (AB Sciex, CA, USA) coupled with an Agilent 1200 high-performance liquid chromatography system (Agilent, CA, USA). The mass spectrometer was operated in the multiple reaction monitoring mode with an electrospray ionization interface. For positive ions ($[M+H]^+$), the source temperature and gas parameters were optimized as follows: ion spray voltage was set to 5500 V, turbo ion spray temperature was set at 500 °C, nebulizer gas (GS1, nitrogen) and heater gas (GS2, nitrogen) were set at 50 L/min, curtain gas was set at 20 L/min, and collision gas (nitrogen) was set at 6 Torr. m/z values of 147.053 → 91.200 (25 eV for collision energy), 165.013 → 137.389 (19 eV for collision energy), 369.155 → 176.900 (37 eV for collision energy) and 237.193 → 194.100 (25 eV for collision energy) were obtained for coumarin, isoeugenol, curcumin and carbamazepine (IS), respectively. Chromatographic separation was carried out using a reversed-phase C_{18} column (X-select C_{18}, 2.1 mm × 100 mm i.d., particle size; 3 μm; Waters, Ireland) at a flow rate of 0.6 mL/min. The mobile phase was composed of 0.1% formic acid in water (A) and 100% acetonitrile + 100% methanol (50:50, *v*/*v*) (B). Gradient elution was performed using the mobile phase at 80:20 (*v*/*v*) ratio of A:B initially. The ratio was changed to 0:100 (*v*/*v*) to 8 min and was returned to the initial composition at 8.1 min, which was then maintained for 10 min. The analytical data were processed using Analyst software (Version 1.7.2, AB Sciex, CA, USA). For sample preparation, a 50 μ L aliquot of plasma sample was deproteinized by adding 150 μ L of acetonitrile containing 0.01 μg/mL of IS. After vortex and centrifuging for 10 min at 12,000 rpm, a 10 μ L aliquot of the supernatant was injected into the column. The coumarin, isoeugenol, curcumin, and IS peaks appeared at 3.7, 5.6, 6.8, and 5.0 min, respectively.

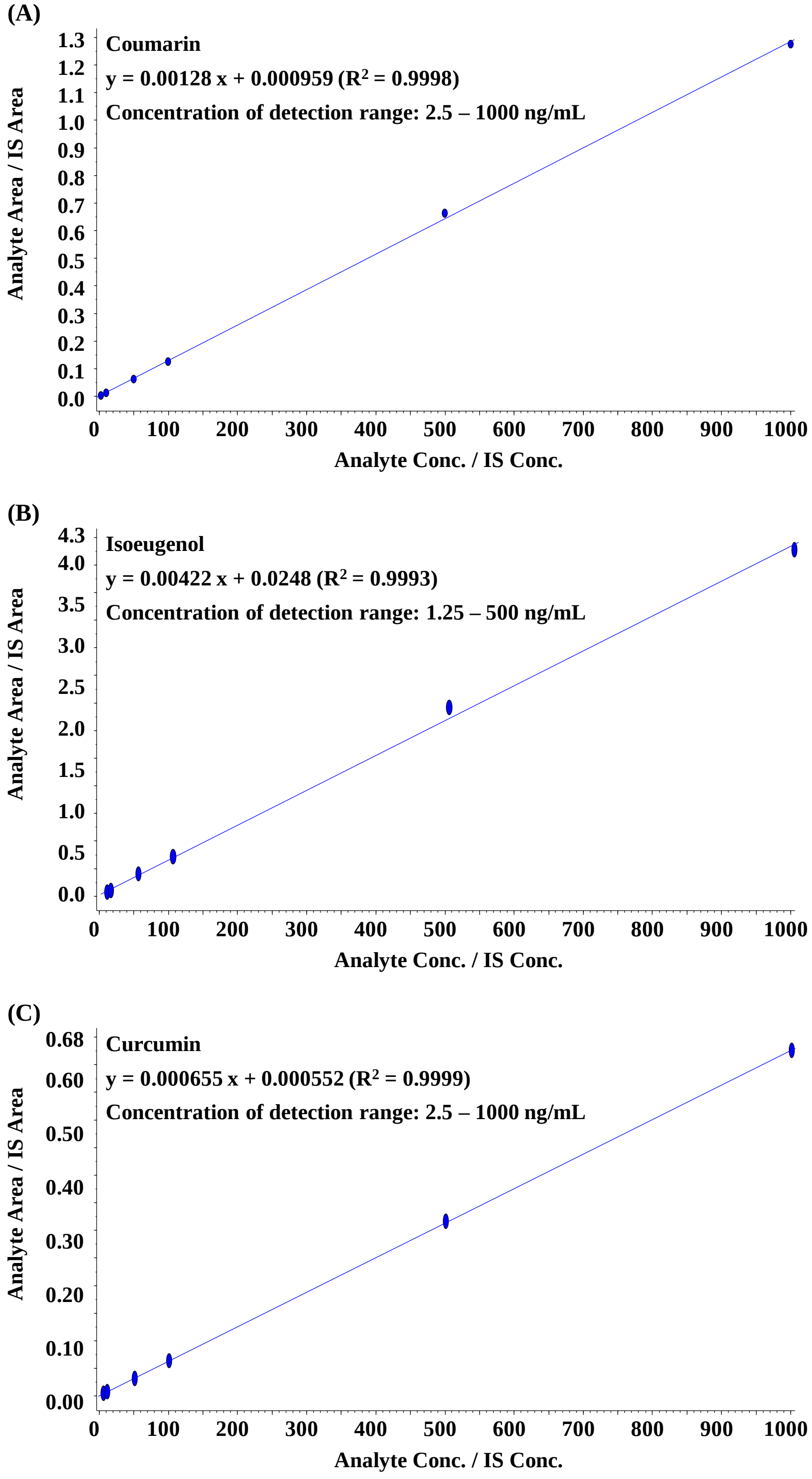

Figure S3. The calibration curves for (A) coumarin, (B) isoeugenol, and (C) curcumin, respectively.
